# GIFT: GRANDMOTHER INITIATIVES IN FAMILY TRANSFORMATION

**DOI:** 10.1093/geroni/igz038.1293

**Published:** 2019-11-08

**Authors:** Carol M Musil, Jaclene Zauszniewski, Alexandra Jeanblanc, McKenzie Wallace, Christina Henrich, Chris Burant, Elizabeth Tracy

**Affiliations:** Case Western Reserve University, Cleveland, Ohio, United States

## Abstract

Little research has been conducted on the development and testing of interventions to support grandmothers during their caregiving experience. Our descriptive and pilot intervention studies provide the foundation for converting a face-to-face resourcefulness intervention into an NIH-funded self-administered, online intervention for grandmother caregivers, to determine its impact on individual health and family well-being. The online intervention provides personal and social resourcefulness training using online video content and structured reflective journaling to reinforce resourcefulness skills compared to reflective journaling. We are testing the resourcefulness intervention in a randomized clinical trial (RCT) with a national sample of 334 grandmothers living with/raising grandchildren, comparing the resourcefulness training protocol vs. unstructured journaling alone. Given the limited interventions available to grandmother caregivers, an intervention that transcends time and place and is available 24 hours a day is expected to bolster the personal and social resourcefulness, which will in turn affect individual and family outcomes.

